# Indole Reverses Intrinsic Antibiotic Resistance by Activating a Novel Dual-Function Importer

**DOI:** 10.1128/mBio.00676-19

**Published:** 2019-05-28

**Authors:** Yan Wang, Tian Tian, Jingjing Zhang, Xin Jin, Huan Yue, Xiao-Hua Zhang, Liangcheng Du, Fan Bai

**Affiliations:** a College of Marine Life Sciences, MOE Key Laboratory of Marine Genetics and Breeding, Ocean University of China, Qingdao, China; b Biomedical Pioneering Innovation Center (BIOPIC), School of Life Sciences, Peking University, Beijing, China; c Department of Chemistry, University of Nebraska—Lincoln, Nebraska, USA; d Institute of Evolution & Marine Biodiversity, Ocean University of China, Qingdao, China; e Laboratory for Marine Ecology and Environmental Science, Qingdao National Laboratory for Marine Science and Technology, Qingdao, China; College of Veterinary Medicine, Cornell University

**Keywords:** *Lysobacter*, antibiotic resistance, *btuD*, indole, vitamin B_12_

## Abstract

Bacterial antibiotic resistance modulation by small signaling molecules is an emerging mechanism that has been increasingly reported in recent years. Several studies indicate that indole, an interkingdom signaling molecule, increases bacterial antibiotic resistance. However, the mechanism through which indole reduces antibiotic resistance is largely unknown. In this study, we demonstrated a novel mechanism for indole-mediated reversal of intrinsic antibiotic resistance in *Lysobacter*. This reversal was facilitated by a novel BtuD-associated dual-function importer that can transfer both vitamin B_12_ and antibiotics. Indole stimulated *btuD* overexpression and promoted efficient absorption of extracellular vitamin B_12_; meanwhile, the weak selectivity of the importer caused cells to take up excessive doses of antibiotics that resulted in cell death. Consistently, *btuD* deletion and G48Y/K49D substitution led to marked reductions in the uptake of both antibiotics and vitamin B_12_. This novel mechanism is common across multiple bacterial species, among which the Q-loop amino acid of BtuD proteins is Glu (E) instead of Gln (Q). Interestingly, the antibiotic resistance of *Lysobacter* spp. can be restored by another small quorum sensing signaling factor, 13-methyltetradecanoic acid, designated *Le*DSF, in response to bacterial population density. This work highlights the mechanisms underlying dynamic regulation of bacterial antibiotic resistance by small signaling molecules and suggests that the effectiveness of traditional antibiotics could be increased by coupling them with appropriate signaling molecules.

## Abstract (precis)

Recently, signaling molecules were found to play a role in mediating antibiotic resistance. In this study, we demonstrated that indole reversed the intrinsic antibiotic resistance (IRAR) of multiple bacterial species by promoting the expression of a novel dual-function importer. In addition, population-dependent behavior induced by 13-methyltetradecanoic acid, a quorum sensing signal molecule designated *Le*DSF, was involved in the IRAR process. This study highlights the dynamic regulation of bacterial antibiotic resistance by small signaling molecules and provides direction for new therapeutic strategies using traditional antibiotics in combination with signaling molecules.

## INTRODUCTION

The increasing emergence of antibiotic-resistant bacterial pathogens poses significant clinical and societal challenges, which provide the impetus for research efforts aimed at understanding the underlying biological mechanisms (1, 2). Multiple mechanisms have been revealed in the past few decades, including the activation of efflux pumps that expel antibiotics, mutations in drug targets, production of enzymes that directly inactivate antibiotics, and biofilm formation (3–9). For example, it is well established that efflux pumps encoded by bacterial genes can confer multidrug resistance, and therefore, the structures and working mechanisms of several multidrug efflux pumps in bacteria have been well characterized (3–5). Meanwhile, the production of specific enzymes results in the inactivation of beta-lactam and aminoglycoside antibiotics by hydrolysis or formation of derivatives (6). Moreover, the formation of bacterial biofilms and their inherent resistance to antibiotics are the root cause of many persistent and chronic bacterial infections (7, 8).

Recent studies have shown that signaling molecules could also mediate antibiotic resistance by promoting the expression of specific genes such as those encoding antioxidant enzymes and efflux pumps (10–14). Here, we focused our study on the interkingdom signal indole that tightens epithelial cell junctions (15, 16). Previous studies have shown that indole, a small molecule that is widely expressed throughout the bacterial kingdom, affects bacterial antibiotic tolerance (17–19). Indole induces the expression of a variety of xenobiotic exporter genes in Escherichia coli (17). Moreover, additional evidence shows that indole reduces persistent formation of E. coli (20–22). YafQ, a specific endoribonuclease, significantly reduced expression of both RpoS and TnaA, which resulted in reduced levels of indole and an increased number of persister cells (20). It was also demonstrated that halogenated indoles inhibited persister and biofilm formation by E. coli and Staphylococcus aureus (21). Phosphodiesterase DosP decreased the activity of tryptophanase, which converts tryptophan to indole, leading to increased persistence formation (22). The intestinal pathogen Salmonella enterica serovar Typhimurium enhances antibiotic tolerance in response to exogenous indole via a process mediated primarily by the oxidative stress response (18). Previous studies also suggest that *S.* Typhimurium effectively received an indole signal produced by cocultured E. coli to enhance its antibiotic tolerance in the intestinal environment (19). By repeated transfer of E. coli in the presence of increasing levels of antibiotic, it was found that indole induces population-dependent antibiotic resistance in E. coli, which suggests that bacterial density may also influence changes in antibiotic resistance caused by small molecules (11). As mentioned above, there are plenty of studies on indole enhancing microbial antibiotic resistance. Relatively few studies have been conducted on the mechanism of indole reducing antibiotic resistance.

*Lysobacter* spp. are common environmental bacteria that have emerged recently as a new source of antibiotics (23–27). For example, heat-stable antifungal factor (HSAF) and analogs from Lysobacter enzymogenes are a group of polycyclic tetramate macrolactams with potent antifungal activity and a distinct mode of action (28). WAP-8294A from L. enzymogenes OH11 and *Lysobacter* sp. strain WAP-8294 is a cyclic lipodepsipeptide compound with promising activity against methicillin-resistant Staphylococcus aureus (27, 29). Another salient feature of *Lysobacter* spp. is their intrinsic resistance to multiple antibiotics (30). However, the molecular mechanism underlying this intrinsic antibiotic resistance is not well understood, possibly because *Lysobacter* spp. produce multiple natural antibiotics. In this study, we describe a phenomenon in which indole reverses the intrinsic antibiotic resistance of *Lysobacter* spp. (indole reversal of antibiotic resistance [IRAR]) by promoting the expression of a novel dual-function membrane importer.

## RESULTS

### Indole reverses the intrinsic antibiotic resistance of *Lysobacter* spp.

**FIG 1:**
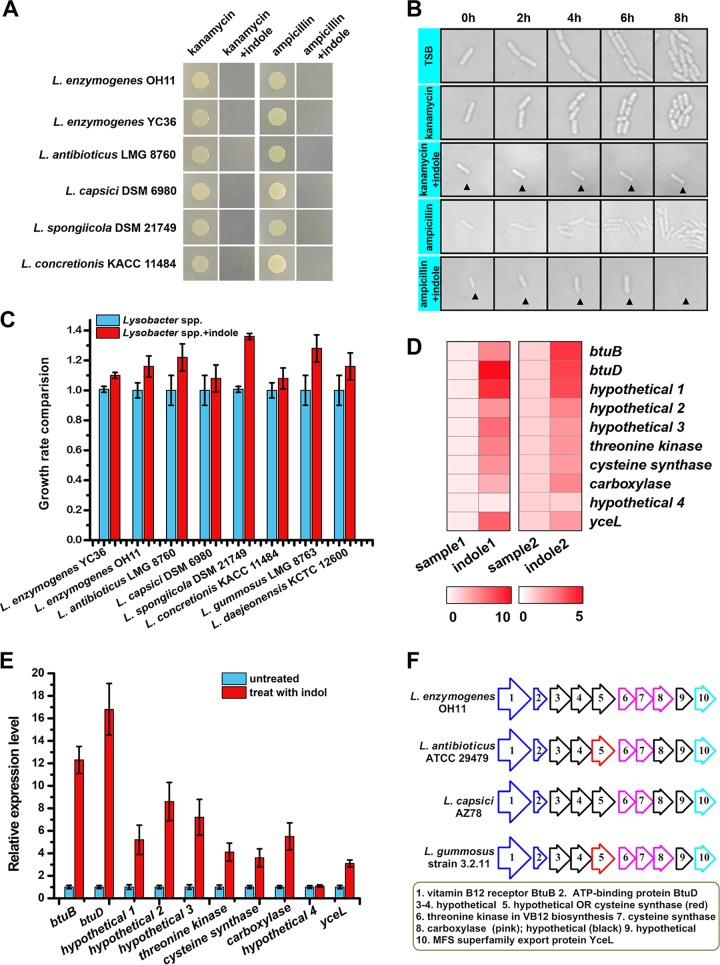
Indole reverses the intrinsic antibiotic resistance of *Lysobacter* spp. and the genome-wide transcriptional profile of *L. enzymogenes* YC36. (A) Indole reduces the antibiotic resistance of *Lysobacter* spp. to kanamycin and ampicillin. Indole was mixed with solid 40% strength TSB medium. The final concentration of indole was 0.5 mM. (B) Dynamic imaging experiment assessing *L. enzymogenes* YC36 growth under different treatments. The concentrations of ampicillin and kanamycin were 100 μg/ml and 50 μg/ml, respectively. (C) *Lysobacter* growth detection after 24-h cultivation in the presence of 0.5 mM indole (right) or absence of indole (left). Indole was added to 40% strength TSB medium at the beginning of cultivation. The results show that indole itself had no toxic effects on cells and slightly promoted growth. The error bars represent the standard deviations for three replicates. (D) Heatmap showing the relative transcript levels of the vitamin B_12_ gene cluster. The scale below the heatmap indicates the fold change of the relative expression level. (E) Real-time PCR assays of the relative expression levels of vitamin B_12_ genes in *L. enzymogenes* YC36. Indole (0.5 mM) was added to 40% strength TSB medium at the beginning of cultivation. (F) The vitamin B_12_ gene cluster analysis of *Lysobacter* spp. The results shown are representative of biological duplicates.

IRAR was observed in all tested species of the *Lysobacter* genus. In traditional plating experiments, the addition of 0.5 mM indole rendered *Lysobacter* spp. sensitive to antibiotic treatment (Fig. 1A). We also monitored the dynamics of bacterial growth under a microscope for bacteria with different treatments. *L. enzymogenes* YC36 cells were able to elongate and proliferate normally with or without antibiotic treatment. However, when both an antibiotic and indole were added to the culture, cells ceased growth or died from cell lysis (Fig. 1B). For a negative control, we determined whether exogenous indole had any toxic effect on bacterial growth. In the absence of antibiotics, indole alone did not result in any adverse effect on cell growth (Fig. 1C), confirming that the combination of indole and antibiotic was responsible for the observed cell death.

### Indole upregulated vitamin B_12_ gene cluster during the process of IRAR.

To explore the biological mechanism underlying IRAR, genome-wide transcriptional profiling of *L. enzymogenes* YC36 treated with or without exogenous indole was performed. Gene profiling showed that 257 genes were upregulated upon indole treatment, while 111 genes were downregulated (*P < *0.005). A number of regulatory genes were upregulated by indole (see Fig. S1 in the supplemental material), including *tetR*, which encodes tetracycline resistance repressor protein, and *luxR*, which encodes HTH-type transcriptional regulator LuxR. The high expression levels of these regulatory genes helped cells to sense exogenous indole signals and regulate the expression of a series of downstream genes. Notably, a cluster of 10 genes, which has been annotated to be associated with synthesis and transport of vitamin B_12_, was significantly upregulated by indole. Expression of the *btuD* gene, which encodes an ATP-binding protein, was upregulated by 10-fold, and the genes flanking *btuD* were similarly upregulated to various degrees (Fig. 1D and E). This 10-gene cluster is common to all species in the *Lysobacter* genus (Fig. 1F). In this cluster, *orf1*, *orf2*, *orf6*, *orf7*, *orf8*, and *orf10* encode the outer membrane vitamin B_12_ receptor ButB, ABC transporter ATP-binding protein BtuD, a threonine kinase involved in vitamin B_12_ biosynthesis, cysteine synthase, carboxylase, and MFS superfamily export protein YceL, respectively. *orf3*, *orf4*, *orf5*, and *orf9* encode hypothetical proteins. BtuD possesses a conserved P loop/Walker A, Walker B, ABC signature domain, and a Switch domain (31). The amino acid sequence of BtuD showed less than 35% identity to any known ABC transporter ATP-binding protein, and the best hit was BtuD from Salmonella enterica subsp. *enterica* serovar Typhimurium (identity of 32.0%) (Fig. S2).

10.1128/mBio.00676-19.1 FIG S1 Heatmap showing the relative transcript levels of indole-activated regulators. Download FIG S1, DOCX file, 0.1 MB. Copyright © 2019 Wang et al. 2019 Wang et al. This content is distributed under the terms of the Creative Commons Attribution 4.0 International license.

10.1128/mBio.00676-19.2 FIG S2 Multiple-sequence alignment of the amino acid sequences of *L. enzymogenes* BtuD, putative BtuD homologs, and other representative BtuD proteins. Download FIG S2, DOCX file, 0.5 MB. Copyright © 2019 Wang et al. 2019 Wang et al. This content is distributed under the terms of the Creative Commons Attribution 4.0 International license.

### IRAR was facilitated by a novel BtuD-associated dual-function importer that can transfer both vitamin B_12_ and antibiotics.

**FIG 2:**
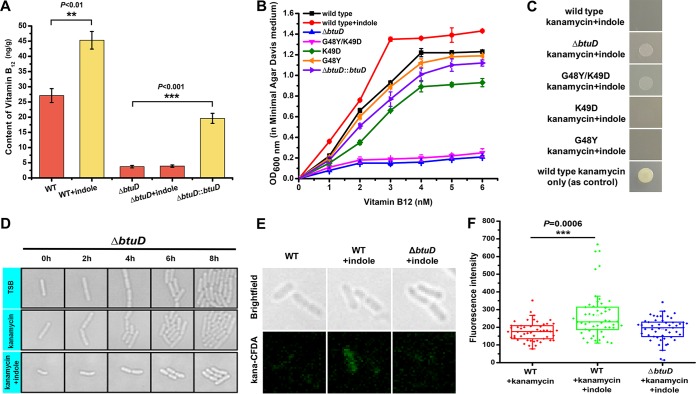
Analysis of the dual functions of *btuD.* (A) Vitamin B_12_ content of the wild-type (WT) strain, *btuD* deletion mutant, and *btuD* complementary strain. (B) The growth speed of the wild-type strain and *btuD* mutants under different vitamin B_12_ conditions (in the presence or absence of 0.5 mM indole). Indole and vitamin B_12_ were added to the medium at the beginning of cultivation. (C and D) The survival states of the wild-type strain and *btuD* mutants under 50 μg/ml kanamycin (in the presence or absence of 0.5 mM indole). (E and F) Fluorescence imaging assay of kanamycin transport by the wild-type strain and Δ*btuD* mutant under different cultivation conditions. The concentration of indole was 0.5 mM. Indole and fluorescent kanamycin were added 5 h before imaging. The results shown are representative of biological duplicates. The error bars represent the standard deviations for three replicates. For statistical analysis, ***, **, and * indicate *P < *0.001, *P < *0.01, and *P < *0.05, respectively.

To confirm that BtuD is responsible for importing vitamin B_12_, we performed a bioinformatic analysis and biochemical assays. We first tested vitamin B_12_ uptake in *L. enzymogenes* YC36 cells with or without exogenous indole. Vitamin B_12_ content was determined by enzyme-linked immunosorbent assay (ELISA) and high-performance liquid chromatography (HPLC). We found that 0.5 mM indole significantly improved the absorption efficiency of vitamin B_12_ (Fig. 2A and Fig. S3). While the Δ*btuD* mutant had weak vitamin B_12_ absorption efficiency, the efficiency of vitamin B_12_ uptake was restored in the *btuD* complementary Δ*btuD*::*btuD* strain (Fig. 2A), which demonstrated that the uptake of vitamin B_12_ was related to the ButD-associated importer. The addition of indole promoted bacterial growth by increasing the efficiency of vitamin B_12_ uptake (Fig. 2B). The Δ*btuD* mutant exhibited very slow growth under vitamin B_12_-deficient conditions. Sequence analysis indicated that residues Gly48 and Lys49 presumably make extensive hydrogen bonding contacts with the phosphate groups of ADP in the P-loop domain (Fig. S4). Next, we induced point mutations in these residues. The mutant with G48Y/K49D double-site substitution showed slow growth, especially under vitamin B_12_-deficient conditions. The mutant with K49D single-site substitution possessed decreased vitamin B_12_ uptake ability and showed slower growth compared to that of wild-type cells. The growth of the G48Y substitution mutant was unaffected by the tested range of vitamin B_12_ conditions (Fig. 2B). Interestingly, the Δ*btuD* strain and the mutant with G48Y/K49D double-site substitution did not show IRAR, while IRAR was observed in G48Y and K49D single-site substitution mutants (Fig. 2C and D). Therefore, we propose that BtuD is a dual-function importer that can transfer both vitamin B_12_ and antibiotics. Indole stimulated BtuD overexpression and promoted efficient absorption of external vitamin B_12_; meanwhile, the weak selectivity of the importer caused cells to take up high doses of antibiotics that resulted in cell death. Consistent with this hypothesis, mass spectrometry showed that indole treatment enhanced the accumulation of antibiotics in cells (Fig. S5). In order to monitor the dynamic entry of antibiotics into cells, we linked the fluorescent probe CFDA-SE (carboxyfluorescein diacetate, succinimidyl ester) to kanamycin to produce a fluorescent antibiotic construct, Kana-CFDA (Fig. S6A to C). Dynamic imaging of Kana-CFDA-SE showed that antibiotics accumulated in indole-treated cells, but the entry of antibiotics into the Δ*btuD* mutant cells was inhibited, which confirmed that BtuD was responsible for cellular uptake of antibiotics (Fig. 2E and F).

10.1128/mBio.00676-19.3 FIG S3 Detection of vitamin B_12_ in *L. enzymogenes* YC36 cells by HPLC. Download FIG S3, DOCX file, 0.3 MB. Copyright © 2019 Wang et al. 2019 Wang et al. This content is distributed under the terms of the Creative Commons Attribution 4.0 International license.

10.1128/mBio.00676-19.4 FIG S4 Structural analysis of Gly48 and Lys49 of BtuD in *L. enzymogenes* YC36. Download FIG S4, DOCX file, 0.5 MB. Copyright © 2019 Wang et al. 2019 Wang et al. This content is distributed under the terms of the Creative Commons Attribution 4.0 International license.

10.1128/mBio.00676-19.5 FIG S5 Mass spectrometry showed that indole accelerated the accumulation of antibiotics in cells. Download FIG S5, DOCX file, 0.1 MB. Copyright © 2019 Wang et al. 2019 Wang et al. This content is distributed under the terms of the Creative Commons Attribution 4.0 International license.

10.1128/mBio.00676-19.6 FIG S6 (A) Synthetic process and core chemical structure of fluorescent antibiotic Kana-CFDA. (B) Verification of the chemical structure of Kana-CFDA by mass spectrometry. (C) Absorption spectrum of Kana-CFDA. Download FIG S6, DOCX file, 0.2 MB. Copyright © 2019 Wang et al. 2019 Wang et al. This content is distributed under the terms of the Creative Commons Attribution 4.0 International license.

### IRAR is common across multiple bacterial species.

**FIG 3:**
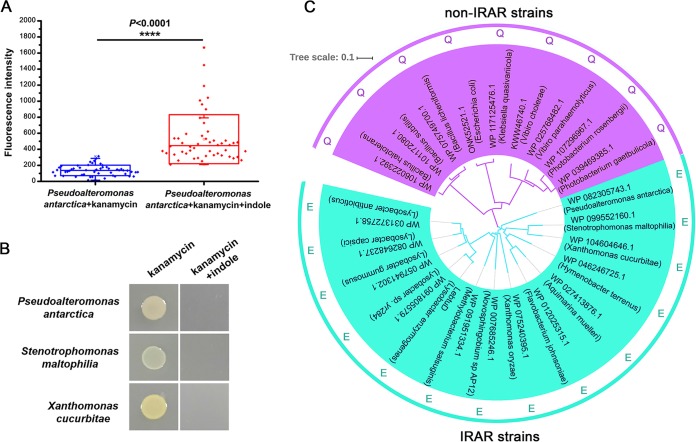
IRAR is observed in a wide range of bacteria. (A) Imaging assay of fluorescent kanamycin transport by Pseudoalteromonas antarctica in the presence or absence of 0.5 mM indole. Indole and fluorescent kanamycin were added 5 h before imaging. (B) Indole reduces the antibiotic resistance of Pseudoalteromonas antarctica, Stenotrophomonas maltophilia, and Xanthomonas cucurbitae. Indole was added to the medium at the beginning of cultivation. The final concentration of indole was 0.5 mM. The OD_600_ of the tested bacteria was set as 0.1. (C) Neighbor-joining tree of BtuD homologs and the sequence characteristics of BtuD proteins from different bacteria.

In subsequent experiments, we found that IRAR is not limited to *Lysobacter* spp. and is shown by several bacterial species. *Pseudoalteromonas* is a common pathogenic bacteria and natural product producer that is intrinsically resistant to multiple antibiotics. Our experiments showed that exogenous indole enabled antibiotics to enter Pseudoalteromonas antarctica cells and accumulate efficiently (Fig. 3A). The resistance of Stenotrophomonas maltophilia, a common clinical pathogenic bacterial species, to a variety of antibiotics makes clinical treatment particularly difficult. IRAR was found to greatly improve the therapeutic effects of antibiotics on S. maltophilia (Fig. 3B). Indole also significantly improved the sensitivity of Xanthomonas cucurbitae, a common pathogenic bacterium in agriculture, to traditional antibiotics (Fig. 3B). Bioinformatic analyses showed that the BtuD proteins of different bacterial strains showing IRAR presented certain obviously similar sequence characteristics. A previous study reported that the glutamine (Q) around the Q-loop of BtuD dominates the surface of the protein that interfaces with membrane-embedded BtuC. However, for IRAR strains, glutamic acid (E), rather than Q, is located around the Q-loop area (Fig. 3C). Although the atomic structure of BtuD has not been resolved, we speculate that this novel feature of the Q-loops of IRAR species changes the manner in which BtuD and BtuC interact.

### *Le*DSF-induced population-dependent behavior is involved in IRAR.

**FIG 4:**
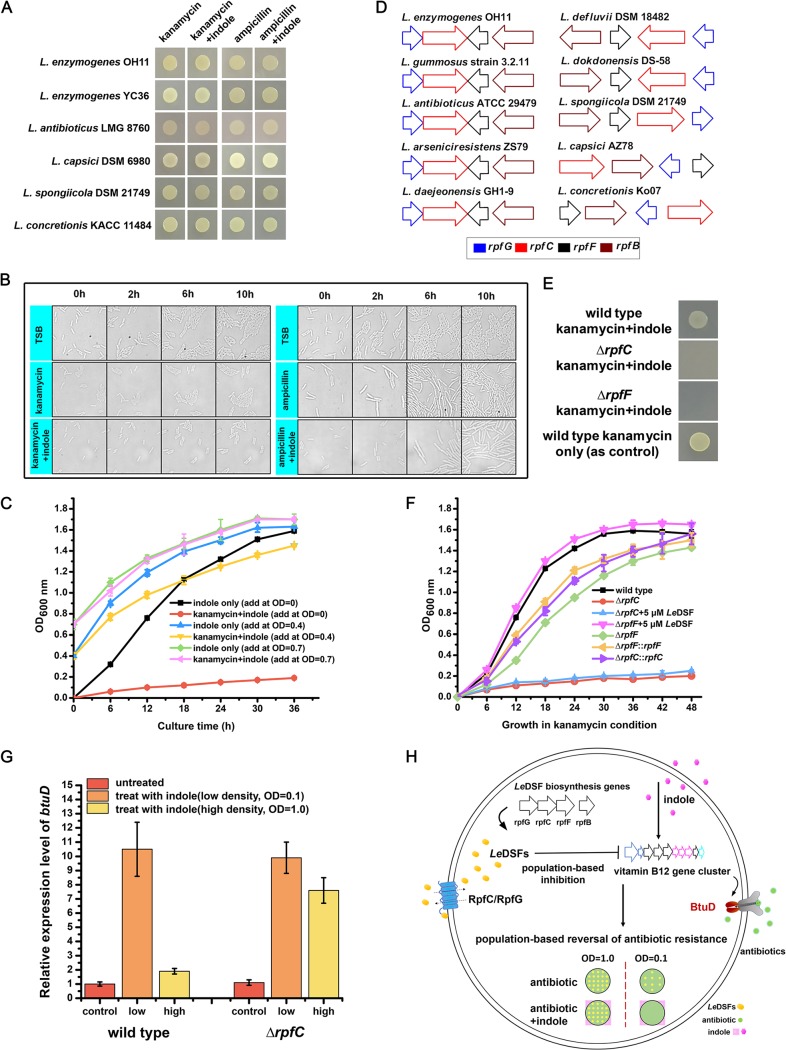
*Le*DSF-induced population-dependent behavior is involved in the IRAR process. (A) The IRAR effect in the stationary-phase (high cell density, OD of 1.0) *Lysobacter* spp. The IRAR process was abolished at high cell density for all tested bacteria. (B) Growth and division of high-density cells (OD of 1.0) under different conditions. (C) Exogenous 0.5 mM indole was added to the medium at different time points (ODs of 0, 0.4, and 0.7, respectively) to detect the IRAR effect. (D) Composition of the *Le*DSF biosynthetic gene cluster. (E) IRAR process detection in the high-density wild-type strain and *Le*DSF-related Δ*rpfC* and Δ*rpfF* mutants. At high cell densities (OD of 1.0), wild-type cells do not show IRAR, but the *Le*DSF deletion mutants show IRAR. (F) Growth curves of Δ*rpfF* and Δ*rpfC* mutants under antibiotic treatment with or without exogenous 5 μM *Le*DSF. *Le*DSF was added at the beginning of cultivation. (G) Relative expression levels of *btuD* in the wild-type and Δ*rpfC* mutant strains with 0.5 mM indole treatment. Expression levels were measured under low (OD of 0.1) and high (OD of 1.0) cell density conditions. The expression level of *btuD* without indole treatment was set as the control. (H) Schematic diagram of IRAR in *Lysobacter.* The results shown are representative of biological duplicates. The error bars represent the standard deviations for three replicates.

Further investigation revealed that the IRAR phenomenon depended sensitively on bacterial population density. When the *Lysobacter* species cell density reached a certain threshold (late exponential phase and stationary phase), indole was no longer able to affect the survival state of *Lysobacter* cells under antibiotic treatment (Fig. 4A). In other words, the intrinsic antibiotic resistance of *Lysobacter* spp. was restored when the cell density was sufficiently high. Microscopic observation revealed that stationary-phase cells grew and divided normally under treatment with indole and antibiotics (Fig. 4B). However, individual cells isolated from the stationary phase could not survive under the same culture conditions (indole with antibiotics) after gradient dilution to a certain threshold. Growth assays in liquid culture confirmed that *L. enzymogenes* YC36 could not grow with antibiotics if indole was added at the beginning of cultivation (OD_600_ of 0). If indole was added at an OD_600_ of 0.4, cells grew slowly, but indole significantly attenuated the survival rate. In contrast, the cultured cells were completely unaffected when indole was supplied at an OD_600_ of 0.7 (Fig. 4C).

To understand the cell density dependence of the IRAR process, we carried out bioinformatic analyses. All of the sequenced *Lysobacter* strains contained a special quorum sensing system induced by *Le*DSF, a diffusible signaling factor-like molecule (Fig. 4D). In a previous study, the chemical formula of *Le*DSF was found to be 13-methyltetradecanoic acid (25). The *Le*DSF biosynthesis gene cluster contains *rpfC*, *rpfG*, *rpfF*, and *rpfB*. The two-component regulatory system encoded by *rpfC* and *rpfG* is responsible for sensing *Le*DSF and triggering subsequent regulatory processes, whereas *rpfF* and *rpfB* encode acyl CoA synthetase and enoyl CoA hydratase, respectively. Coincidently, in the presence of indole and antibiotics, the restored antibiotic resistance of high-density *Lysobacter* was affected if the *Le*DSF-associated genes were deleted. The restored antibiotic resistance was completely lost in the Δ*rpf* mutant (Fig. 4E). The Δ*rpfC* mutant could not survive with antibiotics even when no indole was added, and it could not grow even when supplied with exogenous *Le*DSF. The survival ability of the Δ*rpfF* mutant under antibiotic treatment was decreased in comparison with that of the wild-type cells. However, in contrast with the Δ*rpfC* mutant, supplemental *Le*DSF fully restored the growth of Δ*rpfF* cells under antibiotic treatment. The ability to survive under antibiotic treatment was restored in the *rpfC* and *rpfF* complementary strains (Fig. 4F). The Δ*rpfG* and Δ*rpfB* mutants showed growth similar to that of the wild-type strain, which suggested that the functions of *rpfG* and *rpfB* can be replaced by homologs in the genome. Gene transcription analysis showed that *btuD* was significantly positively regulated by indole during the lag phase and early exponential phase (low cell density, OD of 0.1) in the wild-type strain and the Δ*rpfC* mutant (Fig. 4G). In the Δ*rpfC* mutant, *btuD* expression was increased by 10-fold. However, *btuD* was not obviously upregulated by indole in the wild-type strain during the late exponential phase and stationary phase (high cell density, OD of 1.0), during which *btuD* was upregulated by indole in the Δ*rpfC* mutant (Fig. 4G). On the basis of this evidence, we hypothesize that efficient expression of quorum sensing molecule *Le*DSF beginning in the late exponential phase inhibited *btuD* transcription, thereby inhibiting the IRAR process (Fig. 4H and Fig. S7).

10.1128/mBio.00676-19.7 FIG S7 Relative expression levels of *Le*DSF biosynthesis genes at different time points. Download FIG S7, DOCX file, 0.1 MB. Copyright © 2019 Wang et al. 2019 Wang et al. This content is distributed under the terms of the Creative Commons Attribution 4.0 International license.

## DISCUSSION

Indole signaling is an important means of bacterial communication that has been studied by many research groups. Previous studies reported that indole affected bacterial antibiotic tolerance of E. coli (17, 20–22). In particular, it has been shown that bacterial communication through indole signaling induces bacterial antibiotic resistance by activating stress responses (18). However, compared with the study of indole-induced antibiotic resistance, the mechanism through which indole reduces antibiotic resistance is largely unknown.

In this study, we describe a novel phenomenon in which indole reverses the intrinsic antibiotic resistance (IRAR) of multiple bacterial species. These species were able to elongate and proliferate normally with antibiotic treatment. However, when both an antibiotic and indole were added to the culture, bacterial cells ceased growth or died from cell lysis. Using *L. enzymogenes* YC36 as a model system, we reveal that exogenous indole activates a vitamin B_12_ importer system and improves the absorption of external nutrients. At the same time, exogenous antibiotics are efficiently pumped into the cells and eventually lead to cell death (Fig. 4H). This process explains the IRAR observed in *Lysobacter* cells at low cell density. We report for the first time that the vitamin B_12_ importer system plays a role in xenobiotic transport. Interestingly, our results show that the BtuD homologs of IRAR strains show similar sequence characteristics; glutamic acid (E), rather than Q, is located around the Q-loop area (Fig. 3C). As we mentioned, BtuD in *L. enzymogenes* showed less than 35% identity to the well-studied BtuD from Salmonella enterica subsp*. enterica* serovar Typhimurium. On the basis of the atomic structures of BtuD homologs, we speculate that this novel feature of the Q-loops of IRAR species changes the manner in which BtuD and BtuC interact. It will be interesting to study whether the IRAR process can be abolished if the Q-loop E of IRAR strains is mutated to Q.

When the cell density is high, *Lysobacter* spp. sense nutrition depletion and therefore secrete *Le*DSF, a quorum sensing signal, to ensure the survival of the bacterial population. *Le*DSF effectively suppresses the expression of the vitamin B_12_ importer and thereby reduces the uptake of extracellular antibiotics, allowing cells to survive antibiotic treatment. Quorum sensing is a population-dependent mechanism that enables bacteria to communicate with their neighbor cells and to regulate the levels of expression of multiple genes. Early studies revealed that quorum sensing via *N*-acyl homoserine lactones is closely related to the development of antibiotic resistance and virulence factor production in multiple pathogens (32–34). However, it was unclear whether quorum sensing via *Le*DSF was related to antibiotic resistance. In this work, we demonstrate that *Le*DSF-induced population-dependent behavior is involved in antibiotic resistance. It will be interesting to further characterize whether the IRAR process could also play a role in regulating *Lysobacter*’s population-dependent social activities and its antibiotic production.

## MATERIALS AND METHODS

### Bacterial strains, plasmids, and general methods.

*Lysobacter* strains and the derived mutants were grown in 40% strength TSB medium. Davis minimal medium without methionine was used for the vitamin B_12_ utilization assay (35). The concentration of indole in all experiments was 0.5 mM. The supplemental concentration of *Le*DSF (13-methyltetradecanoic acid) in the experiments was 5 μM. E. coli strains DH5α and S17-1 were used for DNA manipulation and conjugation assays, respectively. Additional bacterial strains and plasmids used in this study are described in Table S1 in the supplemental material. Extraction of plasmids and DNA fragments was performed following the instructions included with the kits purchased from Omega (plasmid mini kit I and gel extraction kit, Omega USA). All molecular manipulations were carried out according to methods described previously (30, 36). Restriction enzymes and molecular biology reagents were purchased from TaKaRa (TaKaRa Bio Group, Japan). PCR primers were synthesized by Tsingke Biological Technology Company.

10.1128/mBio.00676-19.9 TABLE S1 Bacterial strains and plasmids used in this study. Download Table S1, DOCX file, 0.02 MB. Copyright © 2019 Wang et al. 2019 Wang et al. This content is distributed under the terms of the Creative Commons Attribution 4.0 International license.

### Generation of in-frame gene deletion, gene complementary, and site-specific mutants.

To construct vectors for in-frame gene deletion in *L. enzymogenes* YC36, upstream and downstream fragments were amplified using the primer pairs listed in Table S2. Genomic DNA was extracted and used as the PCR template. The upstream and downstream fragments of each gene were cloned into pEX18 to generate in-frame deletion vector pEX18-T. The resulting vectors were transferred into *L. enzymogenes* YC36 according to a method described previously (37), after which target colonies were selected using PCR verification. The confirmed single-crossover colonies were then subjected to double crossover to produce gene deletion mutants. To construct vectors for site-specific amino acid mutants, fragments containing mutation sites were amplified using the primers listed in Table S2. The procedure was identical to that described above for in-frame gene deletion. Plasmid pHmgA-P was used for the gene complementation assay. The target gene was amplified and linked to pHmgA-P to generate vector pHmgA-P-G. pHmgA-P-G was transferred into *L. enzymogenes* by conjugation according to a method described previously (36). All of the mutants were verified by PCR and sequencing verification (Fig. S8A to C).

10.1128/mBio.00676-19.8 FIG S8 (A) Verification of *btuD* deletion mutant strains by sequencing. (B) Verification of G48Y and K49D substitutions by sequencing. (C) Verification of *btuD* complementary strain (Δ*btuD*::*btuD*) by sequencing. Download FIG S8, DOCX file, 0.1 MB. Copyright © 2019 Wang et al. 2019 Wang et al. This content is distributed under the terms of the Creative Commons Attribution 4.0 International license.

10.1128/mBio.00676-19.10 TABLE S2 Primers used in this study. Download Table S2, DOCX file, 0.02 MB. Copyright © 2019 Wang et al. 2019 Wang et al. This content is distributed under the terms of the Creative Commons Attribution 4.0 International license.

### Bioinformatic analyses.

Gene sequences were analyzed by BLAST (http://blast.ncbi.nlm.nih.gov/Blast.cgi). Annotation and bioinformatic analyses were carried out by genome sequencing and EMBOSS (The European Molecular Biology Open Software Suite) (http://emboss.open-bio.org/). ENDscript 2 software was used to compare BtuD proteins (38). Primers for real-time PCR and gene manipulation assays were designed using Primer Premier 5 (39).

### Vitamin B_12_ content analysis.

Vitamin B_12_ content in *L. enzymogenes* was determined by enzyme-linked immunosorbent assay (ELISA) and high-performance liquid chromatography (HPLC). The ELISA experimental procedure was determined according to the instructions of the microbial vitamin B_12_ testing kit from Kanglang Biotechnology Company (Shanghai, China). *L. enzymogenes* was cultured in 40% TSB medium to an OD of 1.0 and transferred to Davis minimal medium with excess vitamin B_12_ for 12 h of cultivation. Due to the weak growth of the Δ*btuD* mutant strain, multiple Δ*btuD* cultures (each with the same volume) were used to ensure a uniform final cell number. The bacterial cells were collected and weighed, and samples of equal weight were used for resuspension and cell fragmentation. The supernatant was removed completely, after which the cell pellet was resuspended with 1 ml ddH_2_O. The content of vitamin B_12_ in the cells was calculated by OD_450_. For the HPLC assay, each bacterial strain was cultured in 40% TSB medium to an OD of 1.0 and transferred to Davis minimal medium with excess vitamin B_12_ for 12 h of cultivation. Cells were collected, resuspended in 50 ml ethanol, lysed, and dried. The precipitate was resuspended in 5 ml methanol. After centrifugation, a 50-μl aliquot of each supernatant was analyzed by HPLC. Pure vitamin B_12_ was used as the positive control. Water/0.1% TFA (solvent A) and acetonitrile/0.1% TFA were used as the mobile phases with a flow rate of 1.0 ml/min. The HPLC program was as follows: 5% solvent B at 0 min, increased to 60% solvent B at 10 min, and reduced to 5% B at solvent 11 min. Vitamin B_12_ was detected at 359 nm.

### RNA extraction, reverse transcription-PCR, and real-time PCR.

*L. enzymogenes* YC36 cells were cultured under different conditions, after which RNA was extracted at various time points using an RNA extraction kit (Omega) according to the manufacturer’s instructions. After the RNA samples were reverse transcribed to cDNA, real-time PCR was performed in a total reaction mixture volume of 20 μl containing 250 nM primers, 10 μl of Eva Green 2× qPCR master mix, 8.5 μl of RNase-free water, and 0.5 μl of 10-fold-diluted cDNA template. 16S rRNA was used as the reference gene. The primers used for qPCR are listed in Table S2. Real-time PCR was performed with a StepOne real-time PCR System (AB Applied Biosystems). The program was designed as described previously (40).

### Transcriptional profiling and analysis.

Transcriptional profiling of *L. enzymogenes* YC36 (with and without indole) was performed by the Biozeron Company in Shanghai, China (PRJNA508225). Total RNA of *L. enzymogenes* (in the absence or presence of 0.5 mM indole) was extracted with TRIzol reagent (Invitrogen). RNA quality was determined and quantified using Bioanalyser 2100 (Agilent) and NanoDrop 2000 instruments, respectively. RNA transcriptional libraries were constructed using the TruSeq RNA preparation kit from Illumina (San Diego, CA). Residual rRNA was removed using the RiboZero rRNA removal kit (Epicenter). Library sequencing was performed on an Illumina Hiseq platform. The raw paired-end reads were trimmed with SeqPrep (https://github.com/jstjohn/SeqPrep) and quality controlled with Sickle (https://github.com/najoshi/sickle). Clean reads were aligned to the reference genome using Rockhopper (http://cs.wellesley.edu/~btjaden/Rockhopper/). EdgeR (41) was used for differential gene expression analysis (https://bioconductor.org/packages/-release/bioc/html/edgeR.html). GO functional enrichment and KEGG pathway analysis were performed using Goatools (https://github.com/tanghaibao/Goatools) and KOBAS (http://kobas.cbi.pku.edu.cn/), respectively. Changes in abundance greater than twofold with *P* values of <0.005 were regarded as significant differences.

### Preparation of fluorescent antibiotic.

First, 50 mg of kanamycin (MW = C_18_H_38_N_4_O_15_S = 582.58, 0.0858 mmol) was added to 10 ml of anhydrous DMF, after which 1 ml of triethylamine was added to the reaction system. Magnetic stirring was carried out under nitrogen protection. Next, 47.8 mg of CFDA-SE [5,(6)-carboxyfluorescein diacetate, succinimidyl ester] was dissolved in 5 ml of DMF. CFDA-SE was added to the kanamycin solution for a 3-h reaction. Finally, thin-layer chromatography detection and high-performance liquid chromatography purification were performed. The chemical structure of Kana-CFDA was verified by mass spectrometry.

### Cell staining for fluorescence microscopy.

For Kana-CFDA (fluorescence antibiotic) staining, cells were collected, washed three times with 40% TSB, and resuspended in 40% TSB buffer. Kana-CFDA was added to a final concentration of 50 μg/ml. For the experimental group, 50 μg/ml Kana-CFDA was added with 0.5 mM indole. Cells were incubated for 5 h in the dark at 30°C with shaking, followed by observation under a microscope.

### Bright-field and fluorescence microscopy.

All images were collected on an inverted microscope (Zeiss Observer Z1). Illumination was provided by solid-state laser (Coherent). The fluorescent signal was collected with an EMCCD camera.

### Time-lapse recording of bacterial growth under a microscope.

We used the FCS2 flow cell system (Bioptechs) to record time-lapse images. Cells were cultured overnight, collected, diluted to a suitable OD value, and washed three times with 40% TSB medium. Next, cells were imaged on a gel pad containing 2% low-melting-temperature agarose. Finally, cells were observed at 30°C under a microscope.

To assess bacterial growth in the presence of antibiotics, cells were resuspended in 40% TSB with ampicillin or kanamycin. To record bacterial antibiotic resistance in the presence of indole, an antibiotic and indole were both added.
