# Supplementary figures and images for: Indole Reverses Intrinsic Antibiotic Resistance by Activating a Novel Dual-Function Importer
Source: mBio. 2019 May 28;10(3):e00676-19. doi: 10.1128/mBio.00676-19 (PMC6538783; doi:10.1128/mBio.00676-19)

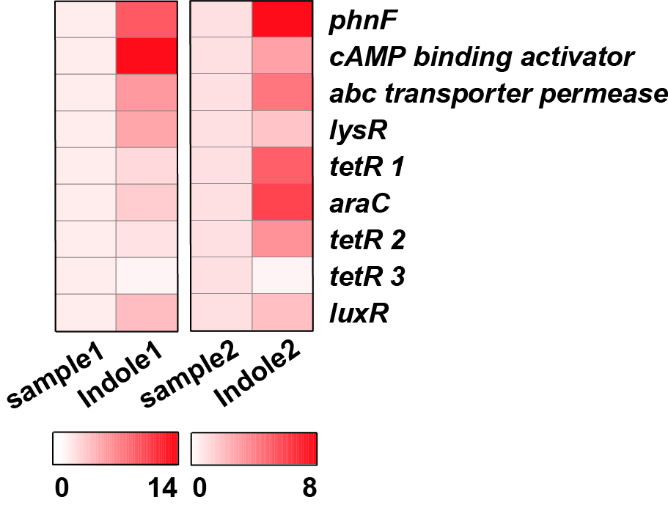


**FIG S1 Heatmap showing the relative transcript levels of indole-activated regulators.**

Supplement: FIG S1 [file mBio.00676-19-sf001.docx]

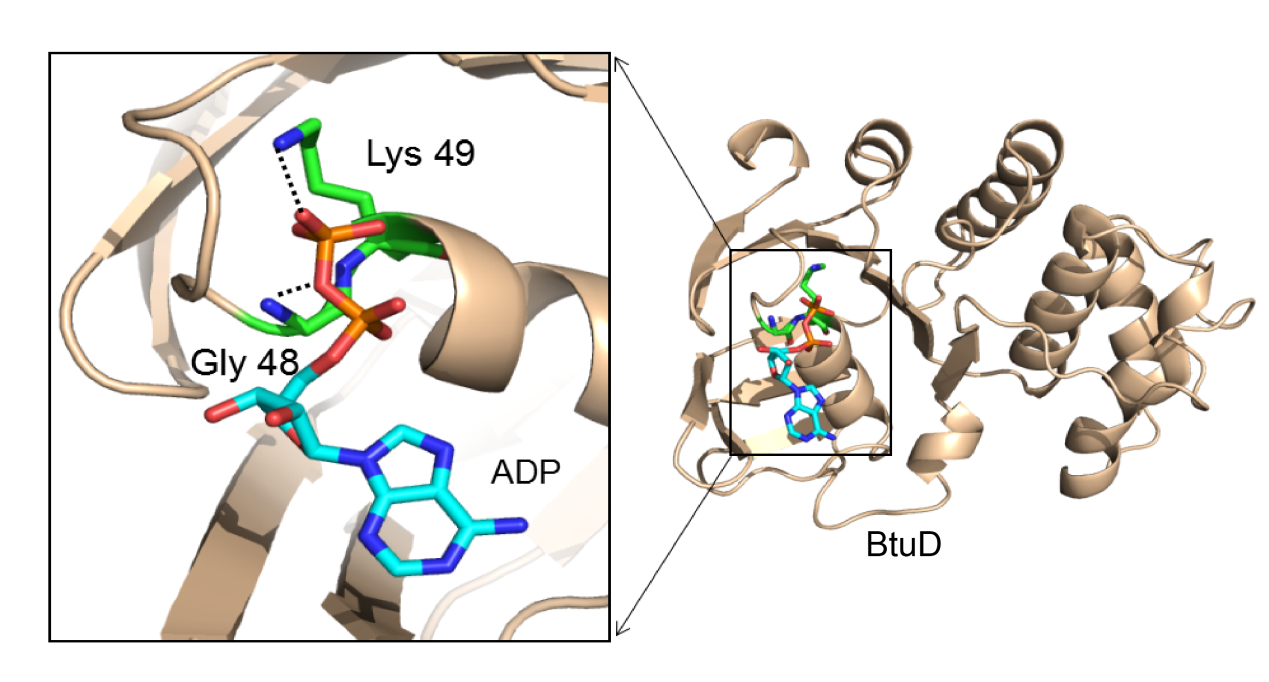


**FIG S4 Structural analysis of Gly48 and Lys49 of BtuD in *L. enzymogenes* YC36.**

Supplement: FIG S4 [file mBio.00676-19-sf004.docx]
