# Supplementary material for: Indole Reverses Intrinsic Antibiotic Resistance by Activating a Novel Dual-Function Importer
Source: mBio. 2019 May 28;10(3):e00676-19. doi: 10.1128/mBio.00676-19 (PMC6538783; doi:10.1128/mBio.00676-19)
Supplement: FIG S2 [file mBio.00676-19-sf002.docx]

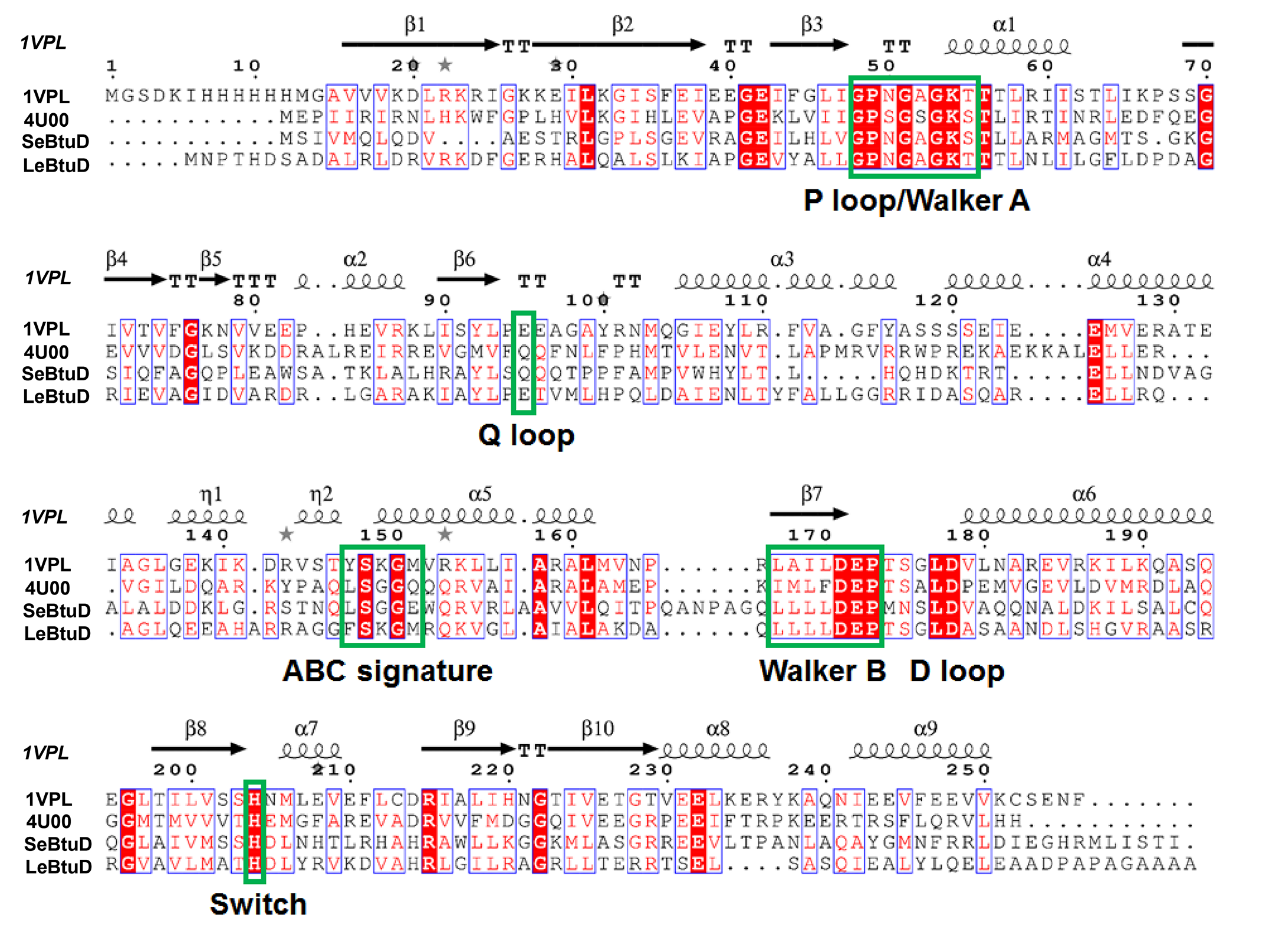


**FIG S2 Multiple-sequence alignment of the amino acid sequences of *L. enzymogenes* BtuD, putative BtuD homologs, and other representative BtuD proteins.** Sequence alignment was performed with the MUSCLE program in the MEGA software package and enhanced by ESPript 3.0. The best hit was BtuD from *Salmonella enterica* subsp. *Enterica* *serovar* *Typhimurium* (identity = 32.0%). Other identified BtuD sequences included 4U00 from *Thermus thermophilus* HB8 (4U00_A) and 1VPL from *Thermotoga maritima* MSB8 (1VPL_A).
