# Supplementary material for: Indole Reverses Intrinsic Antibiotic Resistance by Activating a Novel Dual-Function Importer
Source: mBio. 2019 May 28;10(3):e00676-19. doi: 10.1128/mBio.00676-19 (PMC6538783; doi:10.1128/mBio.00676-19)
Supplement: FIG S3 [file mBio.00676-19-sf003.docx]

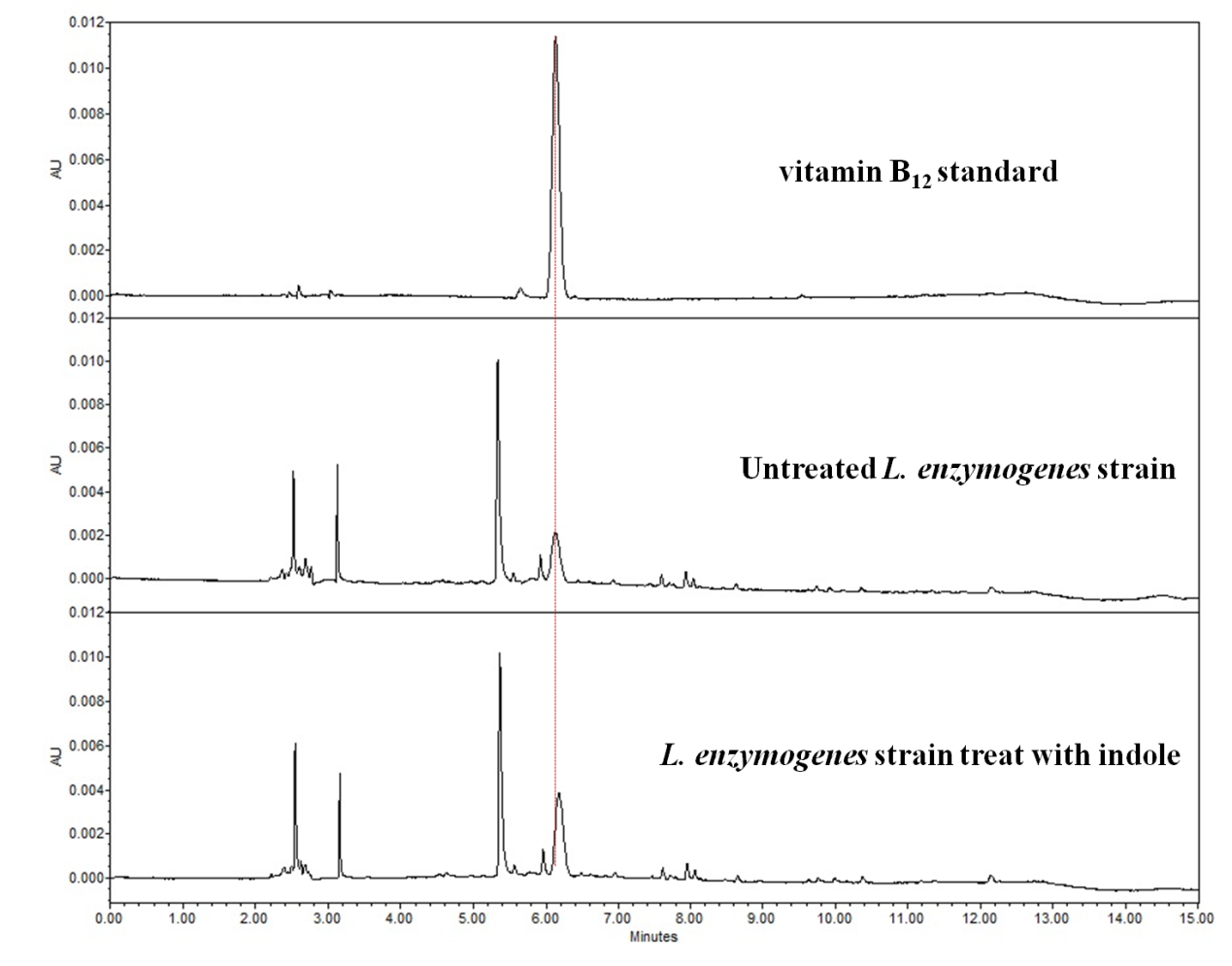
**FIG S3 The detection of vitamin B_12_ in *L. enzymogenes* YC36 cells by HPLC.** HPLC program was as follows: 5% B at 0 min, increased to 60% B at 10 min, and back to 5% B at 11 min. Vitamin B_12_ was detected at 359 nm.
