# Supplementary material for: Indole Reverses Intrinsic Antibiotic Resistance by Activating a Novel Dual-Function Importer
Source: mBio. 2019 May 28;10(3):e00676-19. doi: 10.1128/mBio.00676-19 (PMC6538783; doi:10.1128/mBio.00676-19)
Supplement: FIG S5 [file mBio.00676-19-sf005.docx]

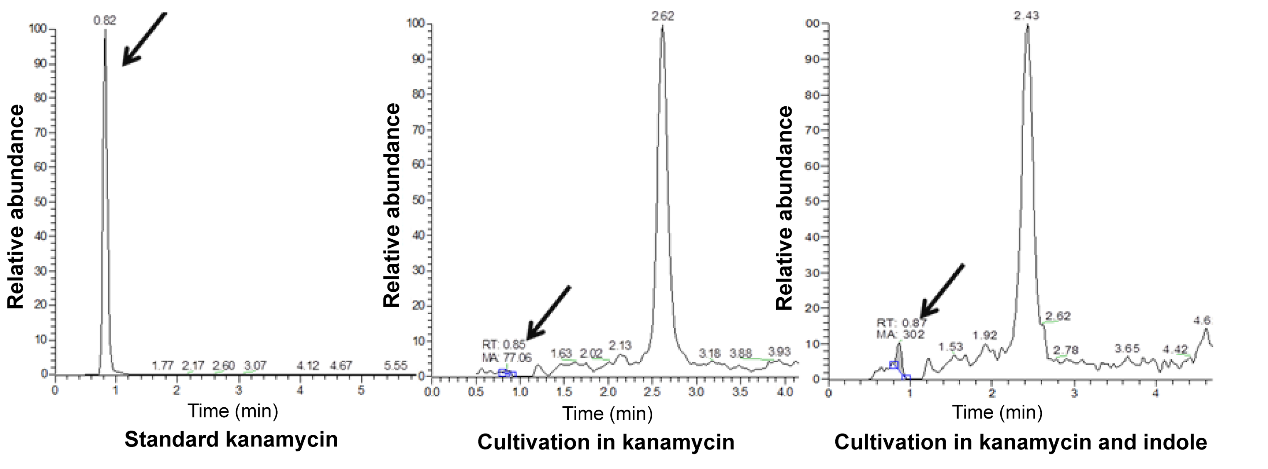


**FIG S5 Mass spectrometry showed that indole accelerated the accumulation of antibiotics in cells.**
