# Supplementary material for: Indole Reverses Intrinsic Antibiotic Resistance by Activating a Novel Dual-Function Importer
Source: mBio. 2019 May 28;10(3):e00676-19. doi: 10.1128/mBio.00676-19 (PMC6538783; doi:10.1128/mBio.00676-19)
Supplement: FIG S6 [file mBio.00676-19-sf006.docx]

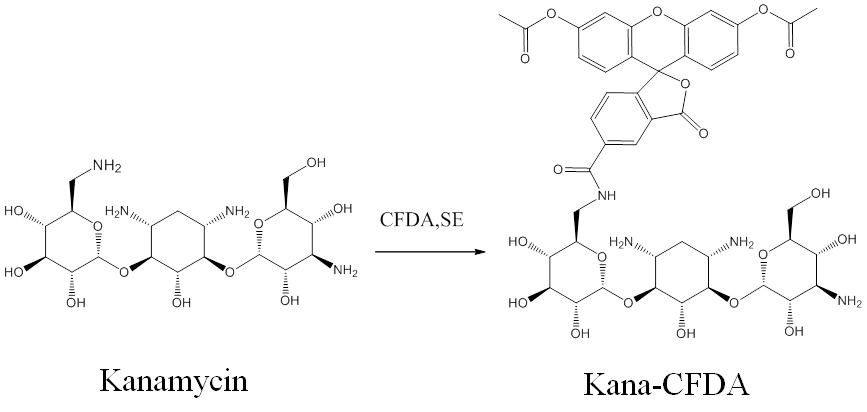


**FIG S6A Synthetic process and core chemical structure of fluorescent antibiotic Kana-CFDA.**

**
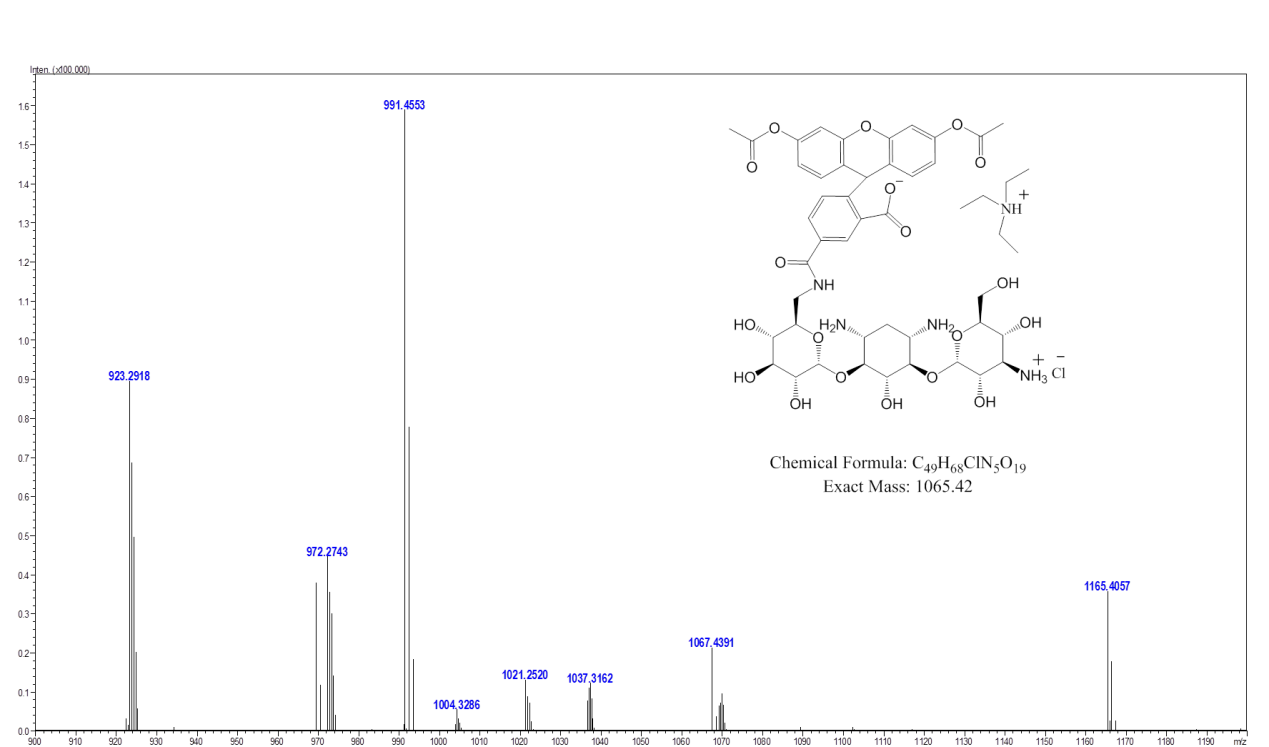
**

**FIG S6B The** **chemical structure verification of Kana-CFDA by mass spectrometry.** The mass was calculated to be 1066.55 for C_49_H_68_ClN_5_O_19_ and found m/z 1067.44 [M + HCl + (C_2_H_5_)_3_N + H] by ESI-MS.

**
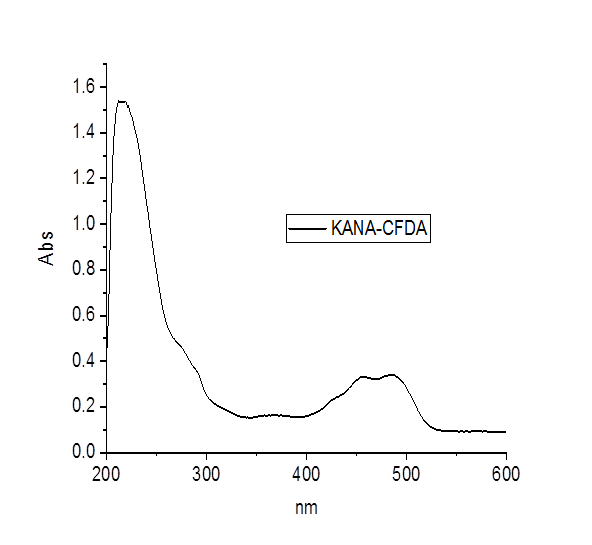
**

**FIG S6C The absorption spectrum of Kana-CFDA.** The result indicated that kanamycin was successfully linked with CFDA.
