# Supplementary material for: Indole Reverses Intrinsic Antibiotic Resistance by Activating a Novel Dual-Function Importer
Source: mBio. 2019 May 28;10(3):e00676-19. doi: 10.1128/mBio.00676-19 (PMC6538783; doi:10.1128/mBio.00676-19)
Supplement: FIG S7 [file mBio.00676-19-sf007.docx]

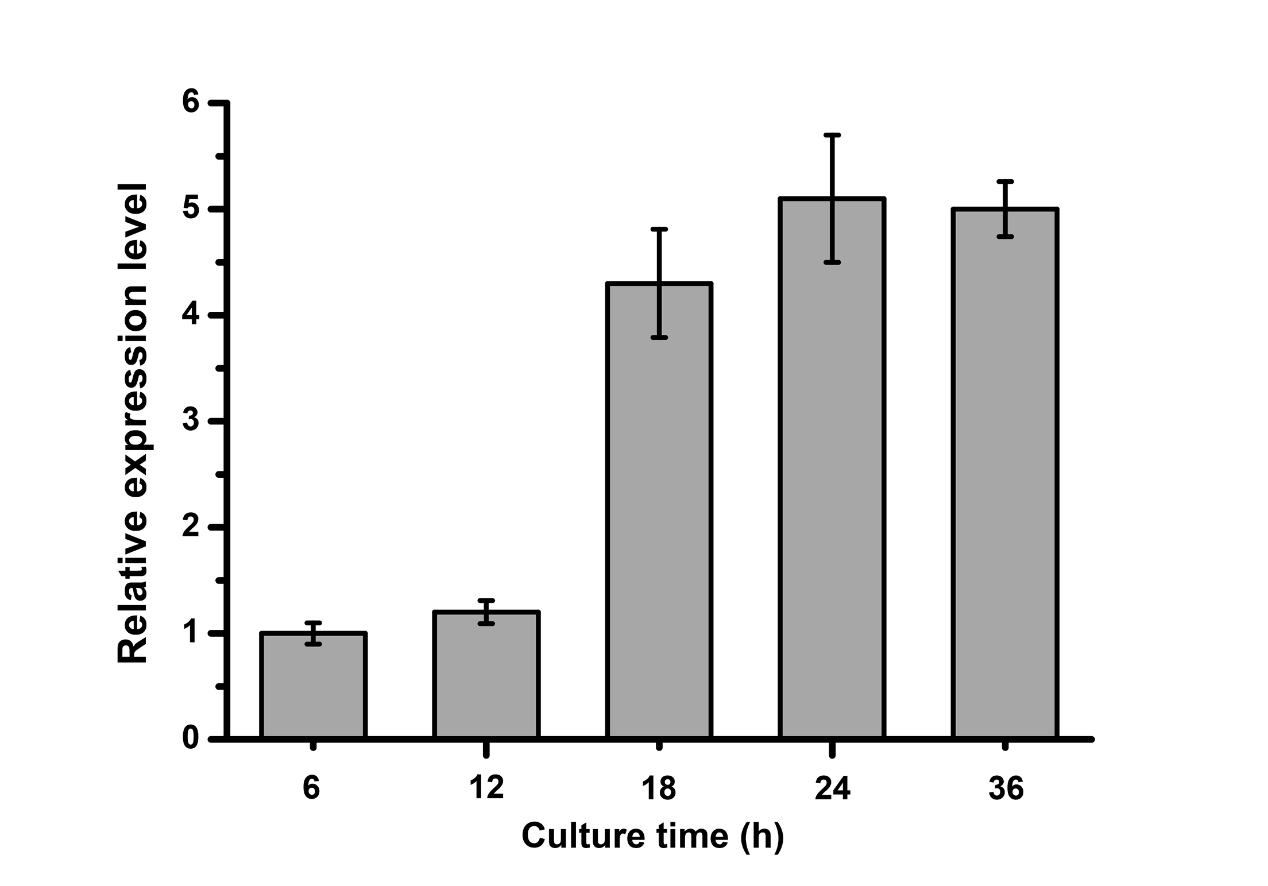


**FIG S7 The relative expression levels of *Le*DSF biosynthesis genes at different time points.** The expression level at 6 hours was set as 1. The error bars represent the standard deviation of three replicates.
