# Supplementary material for: Indole Reverses Intrinsic Antibiotic Resistance by Activating a Novel Dual-Function Importer
Source: mBio. 2019 May 28;10(3):e00676-19. doi: 10.1128/mBio.00676-19 (PMC6538783; doi:10.1128/mBio.00676-19)
Supplement: TABLE S1 [file mBio.00676-19-st001.docx]

**Indole reverses intrinsic antibiotic resistance by activating a novel dual-function importer**

**Running title: Indole-induced antibiotic resistance elimination**

Yan Wang^1,4,5,6^*, Tian Tian^2,6^, Jingjing Zhang^1,6^, Xin Jin^2,6^, Huan Yue^3^, Xiao-Hua Zhang^1,4,5^, Liangcheng Du^3^, Fan Bai^2^*

1. College of Marine Life Sciences, MOE Key Laboratory of Marine Genetics and Breeding, Ocean University of China, Qingdao, 266003, China,

2. Biomedical Pioneering Innovation Center (BIOPIC), School of Life Sciences, Peking University, Beijing, 100871, China

3. Department of Chemistry, University of Nebraska-Lincoln, NE68588, USA

4. Institute of Evolution & Marine Biodiversity, Ocean University of China, Qingdao, 266003, China

5. Laboratory for Marine Ecology and Environmental Science, Qingdao National Laboratory for Marine Science and Technology, Qingdao 266071, China

6. These authors contributed equally.

*Correspondence: [wangy12@ouc.edu.cn](mailto:wangy12@ouc.edu.cn), [fbai@pku.edu.cn](mailto:fbai@pku.edu.cn)

TABLE S1 Bacterial strains and plasmids used in this study

| Strains and plasmids | Relevant characteristics | Source |
| --- | --- | --- |
| Strains | | |
| *L. enzymogenes* OH11 | Wild-type and IRAR strain | This study |
| *L. enzymogenes* YC36 | IRAR strain | This study |
| *L. antibioticus* LMG 8760 | IRAR strain | This study |
| *L. capsici* DSM 6980 | IRAR strain | This study |
| *L. spongiicola* DSM 21749 | IRAR strain | This study |
| *L. concretionis* KACC 11484 | IRAR strain | This study |
| *L. antibioticus* ATCC 29479 | IRAR strain | This study |
| *L. capsici* AZ78 | IRAR strain | This study |
| *L. gummosus* LGM 8763 | IRAR strain | This study |
| *L. daejeonensis* KCTC 12600 | IRAR strain | This study |
| *L. gummosus* strain 3.2.11 | IRAR strain | This study |
| *L. arseniciresistens* ZS79 | IRAR strain | This study |
| *L. daejeonensis* GH1-9 | IRAR strain | This study |
| *L. defluvii* DSM 18482 | IRAR strain | This study |
| *L. dokdonensis* DS-58 | IRAR strain | This study |
| *L. concretionis* Ko07 | IRAR strain | This study |
| *Lysobacter* sp. yr284 | IRAR strain | This study |
| *Xanthomonas* | IRAR strain | This study |
| *Novosphingobium* sp. AP12 | IRAR strain | This study |
| *Stenotrophomonas maltophilia* | IRAR strain | This study |
| *Hymenobacter terrenus* | IRAR strain | This study |
| *Aquimarina muelleri* | IRAR strain | This study |
| *Flavobacterium johnsoniae* | IRAR strain | This study |
| *Pseudoalteromonas antarctica* | IRAR strain | This study |
| *Methylobacterium salsuginis* | IRAR strain | This study |
| *Bacillus subtilis* | No IRAR strain | This study |
| *Bacillus halotolerans* | No IRAR strain | This study |
| *Bacillus licheniformis* | No IRAR strain | This study |
| *Escherichia coli* | No IRAR strain | This study |
| *Klebsiella quasivariicola* | No IRAR strain | This study |
| *Vibrio cholera* | No IRAR strain | This study |
| *Vibrio parahaemolyticus* | No IRAR strain | This study |
| *Photobacterium rosenbergii* | No IRAR strain | This study |
| *Photobacterium gaetbulicola* | No IRAR strain | This study |
| *Escherichia coli* DH5α | Competent cells that can be used for DNA manipulation | This study |
| *Escherichia coli* S17-1 | RP4-2Tc :: Mu-Kn :: Tn7 pro hsdR recA;  host for requiring plasmids; conjugal donor | This study |
| *∆btuD* | *btuD* gene deletion mutant strain that lacks IRAR | This study |
| G48Y/K49D | Mutant strain with Gly48 and Lys49 substitution that lacks IRAR | This study |
| K49D | Mutant strain with Lys49 substitution that retains IRAR | This study |
| G48Y | Mutant strain with Gly48 substitution that retains IRAR | This study |
| *∆btuD* :: *btuD* | The *btuD* complementary strain of *∆btuD* | This study |
| *∆rpfC* | *rpfC* gene deletion strain | From Dr. Du Lab (1) |
| *∆rpfF* | *rpfF* gene deletion strain | From Dr. Du Lab (1) |
| ∆*rpfG* | *rpfG* gene deletion strain | From Dr. Du Lab (1) |
| ∆*rpfB* | *rpfB* gene deletion strain | From Dr. Du Lab (1) |
| *∆rpfC* :: *rpfC* | The *rpfC* complementary strain of *∆rpfC* | This study |
| *∆rpfF* :: *rpfF* | The *rpfF* complementary strain of *∆rpfF* | This study |
| Plasmids | | |
| pEX18 | Gm^r^; oriT^+^ sacB^+^, gene replacement vector with MCS from pUC18 | This study |
| pEX18-T | The plasmid used for gene deletion. pEX18 carrying the *orf* gene from *L. enzymogenes* | This study |
| pHmgA-P | This plasmid contains promoter of HSAF and selection marker. It is used for target gene complementation. | From Dr. Du Lab (2) |
| pHmgA-P-G | The complementary gene was linked to pHmgA-P and formed pHmgA-P-G. This plasmid was used for the expression of target gene. | This study |

**Reference**

1. Han Y, Wang Y, Tombosa S, Wright S, Huffman J, Yuen G, Qian G, Liu F, Shen Y, Du L. 2015. Identification of a small molecule signaling factor that regulates the biosynthesis of the antifungal polycyclic tetramate macrolactam HSAF in *Lysobacter enzymogenes*. Appl Microbiol Biotechnol 99:801-11.

2. Wang Y, Qian G, Liu F, Li YZ, Shen Y, Du L. 2013. Facile method for site-specific gene integration in *Lysobacter enzymogenes* for yield improvement of the anti-MRSA antibiotics WAP-8294A and the antifungal antibiotic HSAF. ACS Synth Biol 2:670-8.
