# Supplementary material for: Indole Reverses Intrinsic Antibiotic Resistance by Activating a Novel Dual-Function Importer
Source: mBio. 2019 May 28;10(3):e00676-19. doi: 10.1128/mBio.00676-19 (PMC6538783; doi:10.1128/mBio.00676-19)
Supplement: FIG S8 [file mBio.00676-19-sf008.docx]

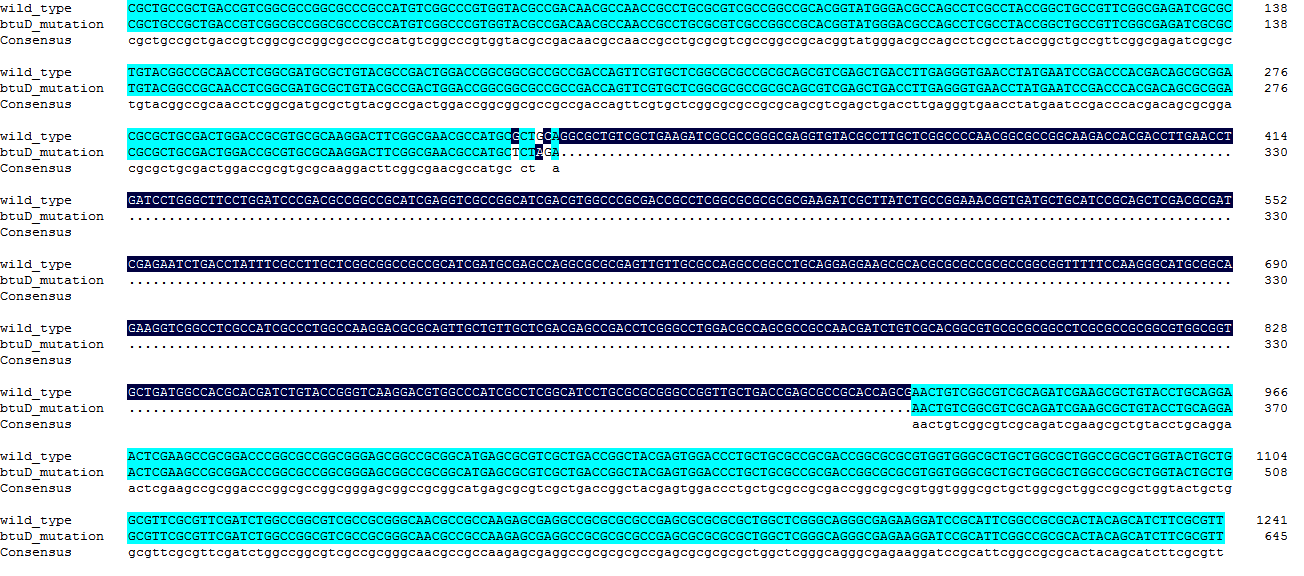


**FIG S8A Verification of *btuD* deletion mutant strains by sequencing.**


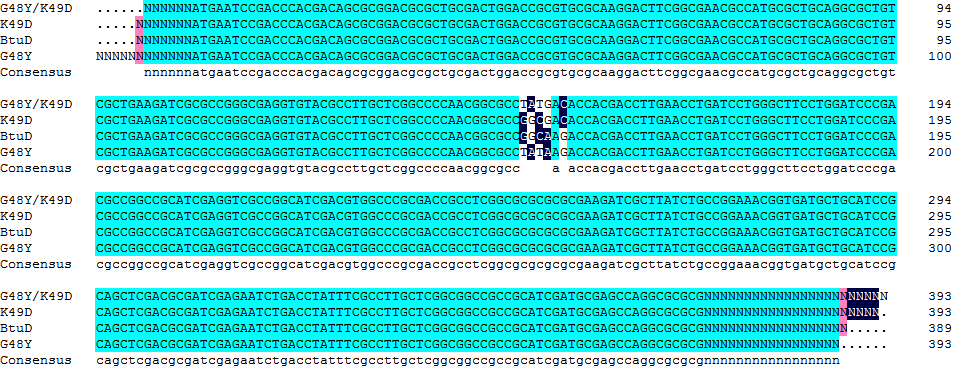


**FIG S8B Verification of G48Y and K49D substitution by sequencing.**


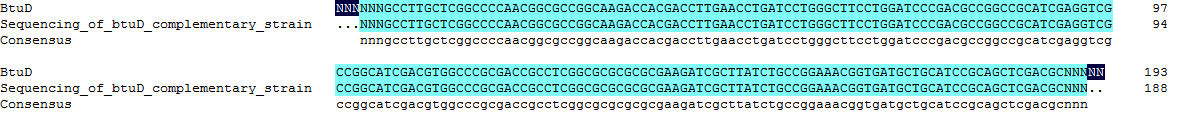


**FIG S8C Verification of *btuD* complementary strain (∆*btuD*::*btuD*) by sequencing.**
