# Supplementary material for: Indole Reverses Intrinsic Antibiotic Resistance by Activating a Novel Dual-Function Importer
Source: mBio. 2019 May 28;10(3):e00676-19. doi: 10.1128/mBio.00676-19 (PMC6538783; doi:10.1128/mBio.00676-19)
Supplement: TABLE S2 [file mBio.00676-19-st002.docx]

**Indole reverses intrinsic antibiotic resistance by activating a novel dual-function importer**

**Running title: Indole-induced antibiotic resistance elimination**

Yan Wang^1,4,5,6^*, Tian Tian^2,6^, Jingjing Zhang^1,6^, Xin Jin^2,6^, Huan Yue^3^, Xiao-Hua Zhang^1,4,5^, Liangcheng Du^3^, Fan Bai^2^*

1. College of Marine Life Sciences, MOE Key Laboratory of Marine Genetics and Breeding, Ocean University of China, Qingdao, 266003, China,

2. Biomedical Pioneering Innovation Center (BIOPIC), School of Life Sciences, Peking University, Beijing, 100871, China

3. Department of Chemistry, University of Nebraska-Lincoln, NE68588, USA

4. Institute of Evolution & Marine Biodiversity, Ocean University of China, Qingdao, 266003, China

5. Laboratory for Marine Ecology and Environmental Science, Qingdao National Laboratory for Marine Science and Technology, Qingdao 266071, China

6. These authors contributed equally.

*Correspondence: [wangy12@ouc.edu.cn](mailto:wangy12@ouc.edu.cn), [fbai@pku.edu.cn](mailto:fbai@pku.edu.cn)

TABLE S2 Primers used in this study

| Primer | Sequence | Purpose |
| --- | --- | --- |
| *btuD*-up | 5′-GCAGGCGCTGTCCTGAAGA-3′ | To construct vectors for gene disruption |
| *btuD*-down | 5′-GCCGCAAAGTGAAGACTCAAGCC-3′ |  |
| *btuD-*1-up | 5′-CGGAATTCCGCTGCCGCTGACCGTCG-3′ | To construct vectors for gene in-frame deletion |
| *btuD-*1-down | 5′-GCTCTAGAGCATGGCGTTCGCCGA-3′ |  |
| *btuD-*2-up | 5′-GCTCTAGAAACTGTCGGCGTCGCAG-3′ |  |
| *btuD-*2-down | 5′-CCCAAGCTTGAACGCGAAGATGCTGTA-3′ |  |
| G48Y/K49D-1-up | 5′-GGGTACCGCCCGCCATGTCGGCCC-3′ | Gly48→Tyr and Lys49→Asp |
| G48Y/K49D-1-down | 5′-GGTGTCATAGGCGCCGTTGGGGCC-3′ |  |
| G48Y/K49D-2-up | 5′-GCCTATGACACCACGACCTTGAACC-3′ |  |
| G48Y/K49D-2-down | 5′-GCTCTAGAGCTCGTCGAGCAACAGCA-3′ |  |
| G48Y-1-up | 5′-GGGGTACCGCCCGCCATGTCGGCCC-3′ | Gly48→Tyr |
| G48Y-1-down | 5′-GGTCTTATAGGCGCCGTTGGGGCC-3′ |  |
| G48Y-2-up | 5′-GCCTATAAGACCACGACCTTGAACC-3′ |  |
| G48Y-2-down | 5′-GCTCTAGAGCTCGTCGAGCAACAGCA-3′ |  |
| K49D-1-up | 5′-GGGGTACCGCCCGCCATGTCGGCCC-3′ | Lys49→Asp |
| K49D-1-down | 5′-GGTGTCGCCGGCGCCGTTGGGGCC-3′ |  |
| K49D-2-up | 5′-GCCGGCGACACCACGACCTTGAACC-3′ |  |
| K49D-2-down | 5′-GCTCTAGAGCTCGTCGAGCAACAGCA-3′ |  |
| *btuD*-C-up | 5′-TCCCCCGGGATGAATCCGACCCACGA-3′ | Used for gene complementation |
| *btuD*-C-down | 5′-CCGCTCGAGTCATGCCGCGGCCGCT-3′ |  |
| *rpfC*-C-up | 5′-TCCCCCGGGATGATCCGCCTGTTGAAC-3′ |  |
| *rpfC*-C-down | 5′-CCGCTCGAGTCAGGGGGAGCGCTCG-3′ |  |
| *rpfF*-C-up | 5′-CGGGATCCATGAGCACCATCGAAA-3′ |  |
| *rpfF*-C-down | 5′-CCGCTCGAGTTACGCGGCCACGGC-3′ |  |
| *btuB*-up | 5′-ACGTCGACAACGGCCTG-3′ | Real-time PCR |
| *btuB*-down | 5′-ATCGCCGTCCAGCGACA-3′ |  |
| *btuD*-up | 5′-AACGGTGATGCTGCATCC-3′ |  |
| *btuD*-down | 5′-AGGTCGGCTCGTCGAGC-3′ |  |
| *hypothetical 1*-up | 5′-CTGGTCGCGTTCCTGCTC-3′ |  |
| *hypothetical 1*-down | 5′-AGCGCCCCGCGCACGCT-3′ |  |
| *hypothetical 2*-up | 5′-TCGACCTGGGCTATGTG-3′ |  |
| *hypothetical 2*-down | 5′-AACACCGCCGGCGCCAG-3′ |  |
| *hypothetical 3*-up | 5′-GAGCTGTTCGCCGACACC-3′ |  |
| *hypothetical 3*-down | 5′-CGGCCTTCGTAGGCCAGG-3′ |  |
| *threonine kinase*-up | 5′-CTCGCCGGCAAGGACA-3′ |  |
| *threonine kinase*-down | 5′-CACCCGCTGGCGGCCGC-3′ |  |
| *cysteine synthase*-up | 5′-AGCCTGCGGGTGGCACGCA-3′ |  |
| *cysteine synthase*-down | 5′-GGAACGGGATCGAACTGC-3′ |  |
| *carboxylase*-up | 5′-TGGGAGCCGGTACGGCGC-3′ |  |
| *carboxylase*-down | 5′-CGGGAACATGTGGCCGT-3′ |  |
| *hypothetical 4*-up | 5′-TGCGCGACCCGCGCACGC-3′ |  |
| *hypothetical 4*-down | 5′-TACCGCCGCGCGCGCGGA-3′ |  |
| *yceL*-up | 5′-CGCTGCTGCTGTTGGCGAT-3′ |  |
| *yceL*-down | 5′-CGAAGTTCAACGCGAAGC-3′ |  |
